# Evidence of free tropospheric and long-range transport of microplastic at Pic du Midi Observatory

**DOI:** 10.1038/s41467-021-27454-7

**Published:** 2021-12-21

**Authors:** S. Allen, D. Allen, F. Baladima, V. R. Phoenix, J. L. Thomas, G. Le Roux, J. E. Sonke

**Affiliations:** 1 Centre for Water, Environment, Sustainability and Public Health (WESP), Department of Civil and Environmental Engineering, University of Strathclyde, Glasgow, G11XJ UK; 2 Laboratoire écologie fonctionnelle et environnement, Université de Toulouse, CNRS, Toulouse, France; 3 School of Geography/Institute for Global Innovation, University of Birmingham, Birmingham, B15 2TT UK; 4 Univ. Grenoble Alpes, CNRS, IRD, Grenoble INP, IGE, 38000 Grenoble, France; 5 Géosciences Environnement Toulouse, CNRS/IRD/Université Paul Sabatier, Toulouse, 3 France

**Keywords:** Environmental sciences, Environmental impact, Atmospheric dynamics

## Abstract

The emerging threat of atmospheric microplastic pollution has prompted researchers to study areas previously considered beyond the reach of plastic. Investigating the range of atmospheric microplastic transport is key to understanding the global extent of this problem. While atmospheric microplastics have been discovered in the planetary boundary layer, their occurrence in the free troposphere is relatively unexplored. Confronting this is important because their presence in the free troposphere would facilitate transport over greater distances and thus the potential to reach more distal and remote parts of the planet. Here we show evidence of 0.09–0.66 microplastics particles/m^3^ over 4 summer months from the Pic du Midi Observatory at 2877 meters above sea level. These results exhibit true free tropospheric transport of microplastic, and high altitude microplastic particles <50 µm (aerodynamic diameter). Analysis of air/particle history modelling shows intercontinental and trans-oceanic transport of microplastics illustrating the potential for global aerosol microplastic transport.

## Abstract (web summary)

Microplastics are found in the environment globally, but their atmospheric transport is not well understood. Here the authors report atmospheric microplastic pollution at the Pic du Midi Observatory, suggesting free long range transport in the troposphere.

## Introduction

Much has been written on oceanic plastic debris since Carpenter & Smith first published the results of their Neuston net tows in the Sargasso sea in 1972^1^. Conversely, airborne microplastic (MP) has only recently been considered. Of the limited studies considering atmospheric microplastics, a large proportion focus on quantifying deposition. The three megacity studies in Paris^2^, London^3^ and Dongguan (China)^4^ found fallout of MP in the order of 175–1008 MP particles/m^2^/day, prompting more cities to begin monitoring their microclimate for microplastic. Monitoring completed in urban and rural Hamburg illustrated similar atmospheric deposition quantities, ~215 MP/m^2^/day and ~396 MP/m^2^/day respectively^5^. However, few studies have attempted to analyse microplastic atmospheric transport. Findings of microplastics in the French Pyrenees Mountains identified daily MP deposition of ~365 MP/m^2^/day at an altitude of 1425 m above sea level. with atmospheric transport of over 95 km^6^. Analysis of London city atmospheric MP illustrated 12–60 km local MP transport (particles and fibres) with long-range transport area of influence of up to 8700 km^2^ (see ref. ^3^). The extensive spatial study of wilderness areas in the USA identified similar atmospheric MP deposition (48–435 MP/m^2^/day)^7^, and noted larger MP particles were more likely regionally sourced (10–1000 km) and rained out while smaller particles were noted as predominantly dry deposition and transported longer distance^7^. Of key importance, the USA study^7^ identified a correlation between dry deposition, regional dust deposition and indices representing broad-scale atmospheric patterns (specifically the southerly jet stream) that point to the potential for free troposphere influence in atmospheric microplastic transport. Building on the USA field findings, Brahney et al.^8^ modelled potential USA atmospheric MP sources, identifying road areas, agricultural soil and oceans as key sources of atmospherically deposited MP in the monitored remote wilderness areas of the USA. Complementary to this, marine studies collecting samples from Shanghai to the Mariana Island (ocean voyage) and the Pearl River to the Indian Ocean identified notable MP (up to 1.37 MP/m^3^) in pumped marine aerosol samples up to ~300 nautical miles offshore (~550 km)^9,10^. HYSPLIT back-trajectory modelling, similar to that used in the Pyrenees and North American wilderness studies, illustrated South China Sea marine aerosol MP particles to potentially have come from Japan, mainland China, Korea^10^ and the Philippines^9^. In addition, the study by Bergmann et al. showed large amounts of MP in snow collected from various sites from the French Alps to Greenland icebergs and Arctic snow^11^. The Bergmann et al. study suggests MP were possibly transported by the wind on the same air currents that carry mercury to the Arctic^12,13^. Furthermore, a global analysis of tyre and brake wear microplastic transport has illustrated that these vehicle-related MP are transported long distances^14^, with 30–34% of tyre/brake wear MP atmospherically transported and deposited in the world’s oceans. The evidence illustrates that MP is present in the planetary boundary layer (PBL), at least at the sites tested, but how far these particles can travel is at least partially dependent on the altitude they can reach within the atmospheric environment. With this in mind, the next logical question to ask is how ubiquitous is MP pollution in our atmosphere, has it reached the free troposphere and what is the extent of free tropospheric MP transport?

Air circulation within the free tropospheric (FT) is a global transport vector for many anthropogenic pollutants including mercury, lead and carbon particulates. The lack of friction from surface topography results in elevated wind speeds and a greater potential for long-distance transport of particle matter. Continental dust that enters the FT has been recorded to circuit the globe, illustrating the extensive transport distances of particulate matter entrained into the FT^15^. If MP is found to occur in the FT, this would suggest that atmospheric MP pollution has the potential to influence the most remote and isolated areas of the globe through FT transport, and that local atmospheric MP pollution may influence a spatial extent far beyond the regional area if the MP are entrained into the FT. With knowledge of FT MP pollution and FT atmospheric MP transport, the presence of MP in the Arctic, Antarctic and remote mountain regions could be explained, and back-trajectory and dispersion modelling of FT MP particles may identify the possible remote area MP pollution sources.

This study presents samples collected at the high altitude long-term monitoring station, Pic du Midi (PDM) Observatory in the French Pyrenees. PDM is defined as a clean station due to its limited influence by local climatic conditions or the environment^16^. PDM has only occasional planetary boundary level (PBL) influence from anabatic (i.e., upslope or valley) winds^17–19^, making it an ideal (and established) site for FT monitoring and analysis^16,17,20^. In this work, we evidence the occurrence, quantity and characteristics of MP at this high elevation, in the FT air masses and its transport pathways. Knowledge of PBL MP pollution illustrates regional atmospheric transport (from key MP emission sources such as cities, agriculture activities, industry, landfills to remote areas^3,5,7,8,14,21^), however, evidence of MP in the FT support long-range, trans-continental and trans-oceanic MP transport.

## Results

### Atmospheric MP found at PDM

**Fig. 1:**
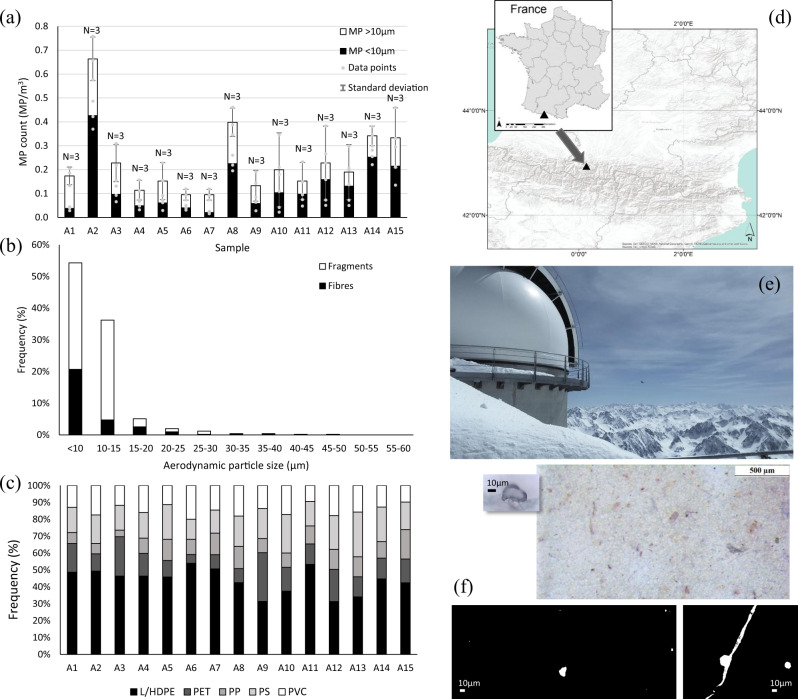
MP characterisation and counts at Pic du Midi (PDM). Microplastic (MP) quantities (MP/m^3^) for the 15 sampling periods (**a**), aerodynamic particle-size distribution (summary of all sampling periods) (**b**) and polymer types (low-/high-density polyethylene (LD/HDPE), polystyrene (PS), polyvinylchloride (PVC), polyethylene terephthalate (PET) and polypropylene (PP)) (**c**) for the 15 samples collect between June 23, 2017 and October 23, 2017 at PDM long-term monitoring station (location (**d**, **e**)). **a** Error bars represent the standard deviation of results (*N* = 3 per sample, *N* = 45 samples overall), with corresponding data points overlaid as grey points. Images of sample filter and selected particles (**f**). The underlying dataset of MP counts is provided in the Supplementary Data. All images in this figure are copyrighted by Steve Allen.

MP samples were collected between June 23, 2017 and October 23, 2017 (15 × 7 day durations). MP fragments or fibres were found in all samples analysed during the summer–autumn monitoring period. Active aerosol sampling presented MP counts of 0.09–0.66 MP/m^3^ at the PDM sampling platform, with an average of 0.23 MP/m^3^ (standard deviation ±0.15) (Fig. 1). In total, 13 of the 15 samples presented MP counts >0.1 MP/m^3^, with 4 samples presenting >0.33 MP/m^3^ (the upper 25th percentile).

Samples show a tentative association between the proportion of MP <10 µm and the fragment content of the sample. Fibres were found to generally occur in greater quantities in the larger MP size fraction, while the smaller size fraction contained a greater proportion of fragment shaped particles (*r* = 0.65, *P* < 0.05). On average, 51% of the MP particles fell within the smaller particle fraction (MP ≤ 10 µm) (21–74% range, standard deviation ±17%), with almost all MP particles characterised as ≤20 µm (aerodynamic diameter) (96%, standard deviation ±0.1). Due to their small size (primarily ≤20 µm), particles were primarily identified as fragments or fibres (70%, 30%, respectively), with fibres defined as presenting a 3:1 length to width ratio^22,23^. The larger MP particles (MP <30 µm) identified were fibres, presenting a maximum aerodynamic diameter of 53 µm. The smallest spectrally characterised particle within this study was 3.5 µm (limit of quantification adopted for this study was 3.5 µm).

MP particles were comprised of polyethylene (LD/HDPE), polystyrene (PS), polyvinylchloride (PVC), polyethylene terephthalate (PET) and polypropylene (PP) (in order of abundance 44%,18%,15%,14%,10%). The polymer type did not correlate significantly with fibre or fragment quantities in the samples or their relative particle-size distribution, with all samples presenting a mix of the five polymer types analysed in the study. This suggests that polymer type or density may not be a key variable influencing MP occurrence at PDM within this study period (however source availability is potentially important).

The wind velocity (monitored at the site for the sampling duration) ranged from 1.4–22.6 m/s (mean 7.9 m/s ± 3.6 m/s), with the stronger winds (≥10 m/s) occurring predominantly from the W to SW (71% of the monitoring period). There is notable intra-sample variation in wind velocity resulting in no clear local wind velocity trend relative to total MP counts. All sample periods present both peak wind velocities for short periods above 10 m/s and calmer wind periods (<5 m/s). Larger MP particles and fibres (MP > 10 µm) show a trend with the maximum recorded wind velocity from the northerly projection (*r*_s_ = 0.79, *P* < 0.05). Stronger wind velocities in general (from any direction) appear to tentatively occur with higher MP >10 µm counts (larger MP) (*r*_s_ = 0.61, *P* < 0.05). This may suggest regional wind velocity could be a transport vector for larger MP at PDM but further long-term monitoring and shorter sampling time step may be necessary to elucidate this potential association.

There appears to be limited correlation or comparable trends between the local meteorological atmospheric conditions monitored (air temperature, relative humidity, precipitation or wind direction) and the total MP particle counts found for the corresponding monitoring periods (*P* ≥ 0.05 for all datasets). MP found in the PDM samples, collected at high altitude, cannot easily be attributed to any clear or specific local influence. The occurrence of MP, especially smaller MP (<10 µm), at PDM may therefore be a result of more complex atmospheric transport, mixing and distal source influence.

### Air mass and particle history and long-distance transport of MP arriving at PDM

Atmospheric transport and particle dispersion models can be used to consider the possible trajectory and dispersion of atmospheric air masses and particulates. The models use reanalysis of meteorological data which allows high confidence in estimating mixing (ground collision/influence) and local weather effects enroute. However, assumptions for particle transport characteristics (density, dry deposition rate, wet removal and scavenging etc.) have to be made as microplastics have limited field validation data. Air-mass back trajectory and particle dispersion analyses were run for 7 days (168 h), long enough to consider possible distal sources and trajectory elevation decline (a commonly used duration adopted in backward modelling for long-distance analysis)^17,24,25^. Back trajectories were created for each hour during the monitoring periods, resulting in 1713 model runs with hourly latitude, longitude and elevation data points. These hourly releases (back trajectories) over the full 24 h provide for the inclusion of any diurnal (day/night) influences on the trajectory elevation (including the change in PBL/FT elevation and associated convection activity). This was complemented by dispersion modelling for the same period (particle dispersed from PDM field location for the active daily sampling period, and backward tracked for the 168 h period).

**Fig. 2:**
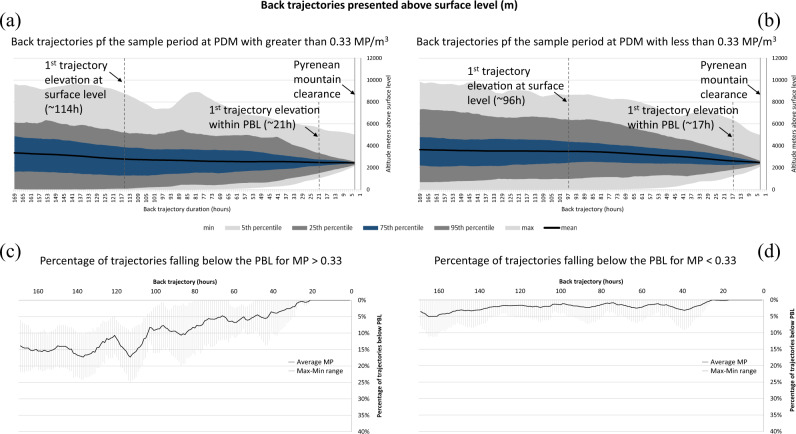
Air mass/particle history from Pic du Midi sample periods. The data are separated to illustrate the higher quantity microplastic (MP) sampling period (MP > 0.33 MP/m^3^, (**a**) and trajectories relating to the lower MP sampling results (**b**). The graphs show the 5th, 25th, 75th and 95th percentile, maximum and minimum elevations above surface level of the modelled back trajectories respectively. **c**, **d** show the average frequency of planetary boundary layer (PBL) mixing with the free troposphere (FT) along the modelled back trajectories for samples with MP > 0.33  MP/m^3^ (**c**) and MP > 0.33 MP/m^3^ (**d**) (average illustrated as the black line, full range of PBL/FT mixing frequency illustrated in light-grey shading).

The back-trajectory and dispersion modelling was overlaid onto the corresponding surface levels to illustrate historic air mass/particle altitude above surface level (ASL) (Fig. 2 and Supplementary Fig. 1). On average, the air mass/particle dispersion backward trajectories maintained an elevation of >2000 m ASL and over the 168 h monitoring period travelled a minimum of 275 km from the PDM sample site.

In general, sample periods with elevated MP quantities (MP > 0.33 MP/m^3^, upper 75th percentile) illustrated atmospheric transport at lower elevations than samples with lower MP particle counts (MP > 0.33 MP/m^3^ average back-trajectory elevation =  2747 ± 373 m ASL; MP < 0.33 MP/m^3^ average back-trajectory elevation = 3276 ± 425 m ASL, MP < 0.13 MP/m^3^ (lower 25th percentile) average back-trajectory elevation = 3123 ± 158 m ASL) (Fig. 2a, b). Correspondingly, the percentage of backward modelled trajectories and dispersion illustrating FT/PBL mixing (the point where the trajectory or particle fell within the PBL elevation above surface level) was notably greater for samples presenting higher MP counts (MP > 0.33 MP/m^3^ average percentage of trajectories falling within PBL = 9% ± 6%; MP < 0.33 MP/m^3^ average percentage of trajectories falling within PBL = 2% ± 1%). The minimum FT atmospheric transport duration and distance (without any PBL influence) prior to reaching PDM was also notably greater for the lower 25th percentile samples (MP < 0.13 MP/m^3^, 887 km (Supplementary Fig. 4) compared to the upper 75th percentile (MP > 0.33 MP/m^3^, 343 km). This tentatively suggests that long-distance FT transported MP forms a potentially greater proportion of MP particles for MP < 0.13 MP/m^3^ samples and more proximal MP (PBL/FT mixing closer to PDM) form a comparatively greater proportion of the MP > 0.33 MP/m^3^ samples. It is noted that while the MP > 0.33 MP/m^3^ samples backward air/particle modelling illustrates a greater frequency of PBL/TF mixing in general, the maximum PBL/FT mixing frequency at any one point in time was <30% and on average 33% of all trajectories (range = 15–57%) included PBL/FT mixing at some point prior to arriving at PDM. This is notably higher (and expresses a greater difference than the general PBL/FT mixing frequency) than the equivalent calculated for the MP < 0.33 MP/m^3^ samples air mass/particle transport histories, where on average <8% (range = 0–18%) of trajectories showed PBL/FT mixing at any one point in time and <12% of all trajectories illustrated any PBL/FT mixing. This reinforces the theory of frequency of PBL/FT mixing being a potentially influential factor of elevated PDM atmospheric MP concentration (MP/m^3^).

When individual samples are considered, only three of the sample periods illustrate modelled backward trajectories meeting surface levels (0 m A.S.L) (A2, A8 and A9) within the modelled 168 h period (a minimum of 114, 122 and 96 h prior to arriving at PDM). Of these, sample A2 and A8 present the highest two atmospheric MP concentrations of the total dataset, while A9 is one of the lower MP concentrations. Five sample periods (A2, A8, A9, A12 and A15; a mixture of elevated and low MP concentration samples) show air/particle histories to be elevated above 50 m ASL for the entire 168 h backward modelling period. This suggests that while proximity to surface level is an important consideration in identifying atmospheric MP sources, the PBL entrainment and PBL/FT mixing is complex and an important atmospheric MP consideration for elevated (FT) observation and sampling sites.

There is a positive correlation between the frequency or percentage of modelled back trajectories falling within the PBL (PBL/FT mixing) and the quantity of MP in the resultant PDM samples. Higher MP concentrations at PDM appear to occur when a greater the number of back trajectories undergo PBL/FT mixing prior to reaching the PDM sampling location (MP counts r = 0.69, *P* < 0.05; MP counts >10 µm *r* = 0.78, *P* < 0.05) (Supplementary Information S5). All samples with the exception of A6 (the lowest MP > 10 µm count sample, 0.066 MP/m^3^) illustrated atmospheric transport that included PBL/FT mixing within the modelled backward 168 h period (free tropospheric transport for greater than 168 h). However, while the frequency of back trajectories with PBL/FT mixing is a potential influence on PDM atmospheric MP concentration, the duration of time the overall trajectories (air mass/particle) spend within the PBL relative to each sample is not significantly correlated to the PDM atmospheric MP concentration. Despite this lack of statistical correlation, there is a visual trend suggesting a possible link between greater average duration of back-trajectory occurrence within the PBL and an elevated MP particle count in the PDM samples (Supplementary Information S5).

Back-trajectory modelling illustrates an average air-mass movement of 4550 km from PDM over the 168 h modelled period (2047–6631 km average trajectory distances for samples A1–A15). The shortest distance travelled (in a straight line from PDM) is 275 km (A1, average trajectory elevation 2775 m ASL) while the longest distance is 10,212 km (A13). Backward modelling of samples with MP < 0.33 MP/m^3^ travelled an average of 4992 km (±1097 km), 1660 km further than samples with MP < 0.33 MP/m^3^. While the overall trend appears to suggest lower atmospheric MP concentration samples at PDM occur in concurrence with greater modelled atmospheric transport pathways, a statistically significant correlation is not evident in this dataset. This is suggested to be due to individual influence of PBL mixing and trajectory elevation above surface-level occurrence along the trajectory rather than the overall atmospheric transport distance.

**Fig. 3:**
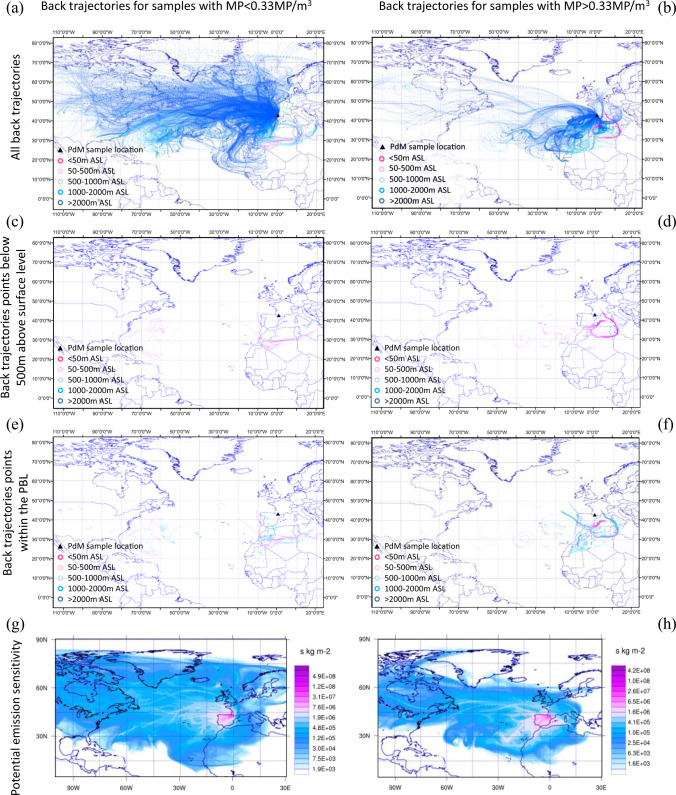
Spatially plotted air/particle histories prior to arrival at Pic du Midi (PDM). Models were run for 168 h in backwards mode for higher microplastic (MP) sample periods (MP > 0.33 MP/m^3^; A2, A8, A14 and A15) (**b**, **d**, **f**, **h**) and the lower MP sample periods (**a**, **c**, **e**, **g**). **e**, **f** present the location of backward trajectory points planetary boundary layer/free troposphere (PBL/FT) mixing points while (**g**) and (**h**) illustrate the relative potential emission sensitivities for the MP < 0.33 MP/m^3^ and MP > 0.33 MP/m^3^ samples, respectively.

The projection of these air mass/particle histories is generally westerly or southerly, across the Atlantic Ocean towards North America or across the Mediterranean Sea towards Northern Africa (Fig. 3a–h, Supplementary Fig. 2 and Supplementary Fig. 3). It is noted that all atmospheric backward modelling from PDM suggest long-distance transport (>100 km)^6^.

All sample periods illustrate an Atlantic Ocean (westerly) trajectory influence. Samples with a higher MP quantity (MP > 0.33 MP/m^3^) show a greater proportion of trajectories over the Mediterranean Sea and Northern Africa (52%) compared to lower MP samples (MP < 0.33 MP/m^3^) (21%) (Fig. 3a, b). Comparatively, backward air/particle history modelling presented a greater proportion of trajectories across the Atlantic Ocean and over North America (53%) for samples with MP > 0.33 MP/m^3^ compared to samples with higher MP particle counts (34%). When lower trajectory elevations are considered (Fig. 3c, d), elevated MP samples were found to have a greater number of trajectory points below 500 m ASL over the Mediterranean Sea and North Africa compared to lower MP samples (MP > 0.33 MP/m^3^ = 73%, MP > 0.33 MP/m^3^ = 55%). Correspondingly, there is a positive trend between the larger particle-size MP findings (MP > 10 µm) and the number of Northern African trajectories in each sample period (Log10(MP > 10 µm) *r* = 0.8, *P* < 0.05). This North African influence alongside the Mediterranean influence is of interest given these areas are identified as presenting high plastic concentrations^26,27^. This mapping suggests that that Northern Africa and the Mediterranean may be areas of potential MP entrainment and influence to the air mass and MP particles at PDM (for the monitored duration).

The elevated MP content in the PDM samples appears to be influenced by both the number of back trajectories that fall within the PBL (frequency) and the location over which the PBL mixing occurs in conjunction with the proportion of the sample that is >10 µm in particle size (Supplementary Data and Supplementary Fig. 5). Sample A2, A8, A14 and A15 (samples with MP > 0.33 MP/m^3^) have an elevated number of modelled back trajectories that fall within the PBL (>10 trajectories points), PBL mixing points over Northern Africa, central and western Europe, the Mediterranean Sea (Fig. 3e, f). The modelling suggests that samples A2 and A8 MP may be transported from Northern Africa, the Mediterranean Sea, Europe and the Atlantic Ocean. Sample A14 appears to primarily have PBL mixing occurring over Northern Africa while A15 back-trajectory modelling suggests Northern African and Atlantic Ocean PBL-mixing points.

Samples with <0.33 MP/m^3^ (A1, 3–7, 9–13) generally have limited the Mediterranean Sea or European PBL-mixing points and a higher proportion of trajectories falling within the PBL over the Atlantic Ocean. The trend between the percentage of trajectories falling within the PBL correlates with both the MP count and MP > 10 µm (*r* = 0.7, *P* < 0.05, see Supplementary Information). While there is notable variance within the PBL-mixing locations, there is also positive trend between the number of PBL-mixing points over land and the number over ocean/sea (MP/m^3^ and land PBL-mixing frequency: *r* = 0.74, *P* < 0.05, *R*^2^ = 0.7; MP/m^3^ and sea PBL-mixing frequency: *r* = 0.81, *P* < 0.05, *R*^2^ = 0.69). This does not suggest land to be a greater MP source influence, with regard to PBL/FT mixing occurrence, and there is a possibility the MP transported into the FT through mixing occurring over the marine environment may be both terrestrial or marine MP^8,28^. This is confirmed by the potential emission sensitivity (PES, Fig. 3g, h) outputs, illustrating elevation particle occurrence over Europe complemented by extensive PES over the Atlantic, Northern Africa and extending to North American continent. Rather than suggesting that terrestrial or marine PBL-mixing locations of are of greater or lesser importance, this tentatively suggests that it is the overall frequency of PBL mixing may be an important influence on atmospheric MP quantities at PDM during this sample period.

## Discussion

Direct comparison to other studies is limited by different minimum size particles analysed and sample collection methodology. Li et al. MP > 5 µm particles in Beijing^29^, Brahney et al. MP > 10 µm particles in USA^7^ and Allen et al. MP > 2.5 µm on French Atlantic^30^ coast are in a similar range as this studies detection limit of MP > 5 µm (LOD 2 µm, smallest µRaman characterised particle 3 µm) (other published studies do not analyse down to this small particle size). Furthermore, many of the published atmospheric MP findings are deposition and therefore not directly comparable to pumped atmospheric samples (Supplementary Fig. 4). It can be seen in Table 1 that the Beijing study found particles several (~5) orders of magnitude greater (5600–5700 MP/m^3^) than this study (0.09–0.66 MP/m^3^). The French coastal air (sea spray) findings were also higher than this present study’s findings, presenting results two orders of magnitude greater than found in this study (pumped coastal air 1.47–19 MP/m^3^). This is understandable given the inner-city sampling location in Beijing compared to oceanic winds and the relative anthropogenic isolation of PDM.

**Table 1:** Summary of published pumped air-mass microplastic (MP) findings.

Location	Microplastic count (MP/m^3^)	Microplastic mean or median counts	MP size range	Predominant size ranges	Environment	Reference
Shanghai, China	0–4.18 MP/m^3^	1.42 MP/m^3^	23 µm–5 mm	<1000 µm	City	^51^
Paris (indoor)	0.4–60.0 MP/m^3^	5.4 MP/m^3^	50 µm–5 mm	20–250 µm	City	^33^
Paris (outdoor)	0.3–1.5 MP/m^3^	0.9 MP/m^3^	50 µm–5 mm	20–250 µm	City	^33^
Sakarya Province, Turkey	116–3424 MP/m^3^	2019 MP/m^3^	50 µm–5 mm	NA	City	^52, 53^
Western Pacific Ocean (Shanghai– Mariana Islands)	0–1.37 MP/m^3^	0.01 MP/m^3^	20 µm–2 mm	318 µm	Offshore marine air	^10^
Surabaya, Indonesia	131–174 MP/m^3^	153 MP/m^3^	~500 µm–5 mm	1000–1500 µm	City	^54^
Asaluyeh County, Iran	0.3–1.1 MP/m^3^	NA	100 µm–1 mm	>100-µm fibres	City	^55^
Beijing, China	5600–5700 MP/m^3^	5600 MP/m^3^	5 µm–2 mm	<20 µm	City	^30^
CSU, USA (indoor)	2.7–17 MP/m^3^	7.9 MP/m^3^	20–>3000 µm	100–300 µm	City	^32^
CSU, USA (outdoor)	0.7–19.6 MP/m^3^	8.3 MP/m^3^	20–>3000 µm	100–300 µm	City	^32^
Atlantic coast, France	0.02–19 MP/m^3^	2.9 MP/m^3^ (onshore)	2.5–300 µm	5–10 µm	Coastal onshore air	^31^
Pearl River Estuary, South China Sea, Indian Ocean	0–0.077 MP/m^3^	0.01 MP/m^3^	58–2252 µm	851 µm	Offshore marine air	^9^
Bushehr port, Iran	0–14.2 MP/m^3^	2.1 MP/m^3^ calm days 10.3 MP/m^3^ dusty days	<2.5 µm	NA	City	^56^
Pic du Midi, France	0.09–0.66 MP/m^3^	0.23 MP/m^3^	3–53 µm	<10 µm fragments 15–20 µm fibres	Remote terrestrial (mountains)	This Study

A summary of all published atmospheric MP findings is provided in Supplementary Fig. 6.

Marine offshore air samples did not include the PM10 or smaller particle fraction focus within the sampling regime and therefore show lower MP concentrations and larger particle-size ranges^9,10^. If only the PDM particle counts above 20 µm and 58 µm are considered (representative of the LOQ for the offshore studies), then PDM results are 0–0.07 MP/m^3^ and 0 MP/m^3^, respectively, (average: 0.01 MP/m^3^ for MP ≥ 20 µm, 0 MP/m^3^ MP ≥ 58 µm). This compares to offshore MP counts of <1.37 MP/m^3^ and <0.077 MP/m^3^ (an average of 0.01 MP/m^3^)^9,10^, illustrating the high altitude PDM results to be within an order of magnitude and slightly lower in MP particle counts than that found in offshore air.

The Californian (CSU, USA) outdoor samples (0.7–19.6 MP/m^3^)^31^ and the Paris outdoor air sampling (0.3–1.5 MP/m^3^) present results within one to two orders of magnitude higher than those found in this study^32^. For both of these studies, this proximity to the PDM findings is primarily due to the analytical size limit being >10 µm MP (LOD/LOQ for these published studies being 20 µm). It is expected that if the smaller MP particle-size fraction was included in the CSU study the MP concentrations in air samples would be substantially higher.

Fibre composition in this study differs from both oceanic^33^ and other atmospheric research in that the predominant material was PE. Marine studies of fibres often cite plastic types as clothing fibres (polyester, acrylic etc.) which is logical given the transport of wastewater from clothes washing to the ocean. It is known that some primary plastics emit fragments during degradation that fit the description of a fibre^34^ as length three times the width. Given the distance from cities that generally have higher fibre counts and that this is a non-marine study, it is a possible explanation for the different compositions.

MP are clearly present in FT atmospheric samples from the monitoring station at PDM and long-range MP transport has been illustrated through both air mass and particle dispersion backward mode modelling. Potential source areas identified include locations across North Africa, Spain, Portugal, France, UK/Ireland and as far as the USA/Canada, as well as the Mediterranean and Atlantic Oceans. Higher MP sample periods appear to be tentatively influenced by western Europe and Northern Africa air-mass trajectories, while lower MP sample periods illustrate trans-Atlantic atmospheric transport and less Northern Africa influence. The variance in particle numbers with similar modelled pathways suggests events further out than the 168 h study period, both spatially and temporally, may be responsible for entrainment. These entrainment mechanisms are an area that requires substantial further research to fully understand the processes at work. At present, there is very limited information on MP atmospheric entrainment specific to the source (e.g. agricultural, industrial, road transport etc.) and the variation of source entrainment relative to the location (different countries with different plastic use and waste management strategies). Therefore, apportionment of atmospheric samples to specific locations and actions difficult and uncertain. Significant future research is needed to quantitatively characterise the location and source-specific atmospheric emissions to advance analysis of source and location contribution of complex atmospheric MP samples.

The mechanics and dynamics of MP atmospheric transport are relatively unknown and un-evidenced. The in-cloud and below-cloud scavenging coefficients have tentatively been considered for tyre and brake wear in a recent study (using statistical assumptions due to lack of physical parameterisation^14^), tyre and brake wear MP particles that are notably more dense than those analysed in the PDM atmospheric study. The wet and dry deposition rate, triboelectric effect, chemical and physical particle interaction in the atmosphere, the influence of humidity, temperature, acidity, precipitation and surface vegetation are all currently unquantified or characterised. Future research is needed to characterise and parameterise MP atmospheric transport dynamics and to identify the key drivers for entrainment, wet and dry deposition and long-distance atmospheric transport. This future parameterisation and transport characterisation will enable more detailed modelling, including particle dispersion analysis, that may provide more detailed insight into atmospheric MP sources and transport.

It should be noted that this study is only for part of the year and seasonal variations are possible, and indeed likely^7^. The one-week sample time step could be shortened to improve model reliability however current plastic pollution levels suggest that longer sample times (≥1 day) are required to obtain a statistically relevant number of particles in remote areas. With increasing production and likely continued mismanagement of waste, it may be possible to shorten the sampling times in future research. The Tisch high Volume sampling system relies on the particle density of dust being 2.65 g/cm^3^, whereas the plastic materials studied are on average around half this density (~1 g/cm^3^). The system does not use a pre-filter to exclude larger material but instead redirects incoming air to reverse its direction (~180° turn), using the material density and kinetic energy to limit the larger particles from reaching the sample filter. Heavier material carries too much kinetic energy to make the turn and collides with a silicone grease pad to which it adheres. The proportion of particles found in this study were greater than the 10 µm normally captured and we believe that this is because the light plastic material can make the turn, avoiding being captured by the grease. It is possible that the types of plastic recorded and/or numbers may be influenced by the Tisch high Volume sampling system and we recommend detailed analysis of how the collection uncertainties and losses specific to this system (laboratory experimentation focused on MP collection relative to total particulate) be conducted in future studies. It is also recommended that sampling be undertaken in the field to compare the total MP to total particulate and sub-sets (e.g. MP < 10 µm be compared to total particulate <10 µm). This study was designed to examine the inhalable <PM10 fraction of microplastic pollution in the FT. The <10 µm counts may be compared to other total particulate atmospheric microplastic studies however we recommend further QA/QC checks be made on the efficacy of the Tisch PM10 filter with regards to microplastics.

Modelling suggests that trans-ocean and trans-continental MP transport may occur. While significant further field and laboratory research is needed to replicate and expand on these findings, the indicative air-mass modelling suggests atmospheric MP to travel extended distances and to occur in the FT. Furthermore, the PBL influence appears important for the MP air-mass concentrations relative to their size alongside the spatial trajectory (over which land mass/marine surface the air history moves) appears to be significant in PDM air-mass MP composition (MP size and overall MP quantity). These findings have implications for remote areas, transporting this new contaminant (and potential pollutant) far beyond its source location. It also indicates a potential risk to environmental and human health due to the possibility of adsorbed chemicals and bacteria/virus being transported long distances prior to deposition in pristine locations and areas vulnerable to exotic chemicals and bacteria/virus.

## Methods

### Study site and sampling method

The field study site is the high altitude Pic du Midi Observatory (PDM) in the French Pyrenees Mountains. The sampling location is an established Global Atmospheric Watch station, latitude and longitude of 42°56’11” N, 0°08’34” E at an altitude of 2877 m above sea level. The atmospheric monitoring platform forms part of the long-term monitoring ongoing at this station^17,35,36^. This station provides extensive aerosol datasets for humidity and chemical compounds including ozone, mercury, carbon monoxide, methane and carbon dioxide^17,37^. The site is defined as remote due to elevation and location. Access to the site is primarily by cable car, with the closest public road ~2.5 km to the south and 1700 m above mean sea level^38^.

This study is designed to determine if MP is present within and transported through the FT. Aerosol sampling was completed using a TISCH high-volume PM10 sampler with a 49833 mm^2^ letterbox format quartz fibre filter membrane over four months of summer/autumn of 2017 (23/06/2017-23/10/2017). Samples were collected over an average of 8.2 days (standard deviation ±1.2 days), with an average pumped air-mass sample volume of 7880 m^3^ per sample (standard deviation ±1206 m^3^) (details provided in the supplementary dataset). Sampling was continuous from 23:00 to 16:00 (21:00-14:00 UTC), with a shutdown period between 16:00 and 23:00 (14:00-21:00 UTC) due to pump noise potentially affecting the telescope observatory activities^18^. A total of 15 samples were collected and analysed for MP content and FT transport plus two field and two laboratory blanks.

All samples were collected on Whatman quartz microfibre (8 × 10 inch) filters with a pore size of 2.2 µm, and all filters were sanitised in a kiln at 530 °C prior to use to ensure filters were clean of contaminants. Filters were handled and prepared under a class 100 flow hood. Filters were then placed in the TISCH high-volume sampler and the air suction pump activated of the monitoring period, with automatic shutdown each day between 16:00 and 23:00. At the end of each sampling period, samples were collected and placed in sanitised aluminium foil envelopes and stored in the dark under refrigeration conditions (−20 °C). Cotton laboratory coats and nitrile gloves were worn at all times when manipulating sample filters and equipment.

### Analytical procedure for the detection of MP particles

Three 30-mm diameter circular areas (stamp outs, randomly selected) of the quartz filter were analysed for MP. There is a necessary assumption that the TISCH high-volume sampler collects MP evenly across the filter in a similar manner to Hg and PM10 particulates. The three 30-mm diameter sub-samples were removed from the quartz filter sheet using a kiln sterilised stainless-steel circular stamp in a positively pressurised room and under a class 100 flow hood. The sub-samples, for MP samples and field blanks, were then placed in kiln sterilised aluminium envelopes and stored in a glass container in dark, refrigerated conditions.

MP samples (and field blanks) quartz filters were flushed with 250 ml MilliQ (18 MΩ.cm) ultrapure water into sterilised borosilicate glass test tubes. Samples (material + MilliQ) were then filtered onto aluminium oxide filters (Whatman Anodisc 0.2-µm pore, 25 mm diameter) using borosilicate glass vacuum filtration. The filters were vacuum air-dried (sterilised aluminium and glass test tube caps were used to minimise air MP contamination) and placed in borosilicate glass capped dishes prior to µRaman analysis.

µRaman analysis was completed for each sample using a Horiba XploraPlus (50–3200 cm^−1^ with a 1.5 cm^−1^ resolution, confocal imaging accuracy 0.5 µm with motorised *X-Y* stage). Each filter was analysed for total plastic presence using the 785 nm laser (spatial resolution of 1 µm) and 200–2000 cm^−1^ Raman shift range. Filters were analysed using the cross-section analysis method presented in Huppertsberg and Knepper^39^. Spectra were collected using an acquisition time of 15 s and 10 accumulations, maximum of 25% power (filter) (general settings: grating of 1200 gr/mm and 50 µm split, modified to achieve effective spectra results as necessary during analysis)^6^. µRaman analysis was completed using Spectragryph 1.2.14 to manage Raman spectra and open access Raman plastic library SLOPP and SLOPPE^6,40,41^. Particle counts and shape identification were identified using FIJI (ImageJ 1.52p)^42^ to define the fibre and non-fibre particle shape, particle size (including aerodynamic diameter)^6,43^.

Filters were then analysed by confocal microscopy and imaging to provide a secondary or validation of particle count (actual reported particle counts are µRaman results) and particle dimension dataset similar to the visual method defined in Brahney et al.^7^. Due to the small size of the particles in the sample dataset fibres were defined as elongated particles with a length to width ratio of 3:1. All other particles are counted as general fragments (a compilation of fragments, films and foams), with no definition provided between fragments, films and foams to limit potential errors in visual identification. Fragment and fibre size and the count was completed using ImageJ (FIJI)^44^ covering the full extent of the 25-mm aluminium oxide filters.

### Contamination and procedural blanks

All MP samples collected at the PDM monitoring station were collected in a manner to minimise contamination. Cotton clothing and laboratory coats were worn at all times when manipulating samples, with all sample filtration material, storage envelopes (aluminium) and manipulation equipment (stainless-steel tweezers, spatulas etc) sterilised through kiln heating (300 °C) to minimise contamination. Field (full procedural) blanks were made during this sampling campaign, on August 28, 2017 and October 23, 2017, following the same protocol for preparation, installation and storage for the MP sample filters. Field blanks (2) were created and analysed following the same protocol as the MP samples and laboratory samples, with an average of 8 particles in field blanks (full procedure) and 3–4 in lab blanks. Fielded blanks represent <30% of the counts (further details available within the Supplementary Dataset). Laboratory blanks (2) were created during the sample preparation period, following the full protocol for MP sample preparation and analysis. In line with all previously published MP analyses to date, the MP counts found on the atmospheric samples were reduced by the MP found in the field and procedural blanks, providing the resultant sample counts^6^.

### Air-mass history and particle dispersion modelling

Hybrid Single-Particle Lagrangian Integrated Trajectory (HYSPLIT) modelling^24^ undertaken using HYSPLIT version 4 (April 2018). Analysis was undertaken to consider the history (back trajectory) of atmospheric air masses carrying particulate material to establish the probable direction of MP transport to PDM, the associated trajectory and therefore possible source areas^45^. Modelling of air-mass particulates was considered appropriate due to the limited knowledge on atmospheric MP dynamics and the small size of the sample particles. Initial analysis to examine air-mass particle history (trajectory) spatial extents and elevation were undertaken using ECMWF ERA-Interim analyses and presented in Fig. 3 (and Supplementary Figs. 2 and 3). HYSPLIT was run in backwards mode, models were run for 168 h from the PDM sampling platform location, 100 m above surface level (~3000 m above sea level). A new back-trajectory model was run for each hour within the sampling period, resulting in 1713 modelled air-mass particle history trajectories. Trajectory spatial and elevation information were extracted and mapped using Esri (ArcGIS 10.4).

The flexible particle dispersion model (FLEXPART) version 9.02 was completed to consider the complex PBL/FT mixing, atmospheric turbulence and the influence of the PBL (potential MP from the PBL and relative surface areas) on the air-mass MP content along the full trajectory towards PDM^46–49^. FLEXPART was used to identify the potential source regions and air-mass history for observed MP particles^47,48^). The model was run in backward mode for 7 days (168 h) for each hour of sampling from 23rd June to 23rd October 2017. Periods between 16:00 and 23:00, when the samplers were closed, were excluded from FLEXFART modelling. For each hour of sampling, particles were released continuously at the location of the sampling site in the Pyrenees (3000 m ASL). The model was driven with 3 hourly operational meteorological/ ERA-Interim analyses from the European Centre for Medium Range Weather Forecasts with 0.5˚ × 0.5˚ resolution and 60 vertical layers. The model simulates the trajectories for the particles which represent the transport by three-dimensional wind fields, turbulent, diffusive transport. A passive air tracer was used due to the limited information on MP atmospheric dynamics and in/blow cloud removal rates. Model results were presented as a spatial distribution of the source-receptor sensitivities (SRS) or simply potential emission sensitivities (PES), which are represented as and are related to the particles’ residence time in the output grid cells. The values of this model parameter can be interpreted as spatial information indicating those areas where a larger or smaller proportion of the air at the respective site came from.

### Data analysis

Statistical analysis of µRaman spectroscopy to estimate the MP count per analysed filter and total TISCH high-volume filter relative to the air mass pumped through the filter was undertaken through simple extrapolation. MP identified in the µRaman sampled areas (~20% of each filter) were used to provide a polymer specific MP count and used to confirm visual and ImageJ MP counts for the 30 mm diameter sub-samples (three sub-samples per sample period, analysed as replicates to create a representative MP count). The representative air mass passing through each 30-mm-diameter sub-sample was calculated from the recorded total TISCH high-volume air mass sampled per sample period (6624–11799 m^3^, detailed in the supplementary dataset) (total filter area 0.0516 m^2^). MP counts were reported relative to 1 m^3^ of air mass sampled for each sample period (A1–A15).

Aerodynamic particle diameter was calculated for all particles following the standard equation:

1 $${D}_{a}\approx {\left({d}_{c}{{{{{\rm{ln}}}}}}2\beta \right)}^{1/2}{D}_{c}$$

where Da is the aerodynamic diameter, dc is the density of the particle specific to the polymer type, β is the aspect ratio of the particle and Dc is the cylindrical diameter of the particle^3,50^.

Local meteorological data was publicly available through the P2OA database, generally in hourly format. The sample MP counts, polymer sample compositions and fibre:fragment content of each sample were analysed relative to the mean, mode, maximum, minimum and variance of each sample period (ensuring shutdown periods were not included). Independence and correlation for parametric and non-parametric datasets were considered (as appropriate to the individual dataset) to identify any statistically significant trends (Pearson or Spearmen *U* tests) and the MP dataset transformation by Log10 was used where datasets were non-parametric to provide additional insight.

HYSPLIT back-trajectory elevations were extracted from individual HYSPLIT model runs and analysed to define the 25th, 50th, 75th, maximum and minimum elevations. Extracted HYSPLIT 3D datasets were converted to ArcGIS (ESRI) shape files and used to calculate trajectory distances from PDM relative to elevation and trajectory duration (Fig. 2 and Supplementary Figs. 1 and 2). FLEXPART dispersion model results were extracted to present emission sensitivity (PES), trajectories, mixing depth elevations and particle content (%) relative to time and location. The PBL/FT mixing points were extracted from these datasets and mapped relative to the respective sample period using ArcGIS (ESRI) (Fig. 3a).

### Reporting summary

Further information on research design is available in the Nature Research Reporting Summary linked to this article.

## Supplementary information

Supplementary Information

Description of Additional Supplementary Files

Supplementary Dataset 1

Reporting Summary

## Data Availability

The microplastic data generated in this study have been provided in the Supplementary dataset (xls) provided with this manuscript (MP counts, particle-size distribution, shape and polymer type relative to the sample periods). The meteorological dataset (air temperature, wind direction and speed, atmospheric pressure and precipitation) of the Pyrenean Platform for Observation of the Atmosphere (P2OA) was accessed (and is freely accessible) online via http://p2oa.aero.obs-mip.fr. All data needed to evaluate the conclusion in the paper are present in the paper and/or Supplementary Materials. All samples, analysis, publication and ownership of data are free from legal entanglement or restriction of any sort.

## References

[1] Carpenter EJ, Smith KLJ (1972). Plastics on the Sargasso Sea surface. Science.

[2] Dris, R., Gasperi, J. & Tassin, B. Sources and fate of microplastics in urban areas: a focus on Paris Megacity. In *Freshwater Microplastics The Handbook of Environmental Chemistry* (eds Lambert, S. & Wagber, M.) 69–83 (Springer, 2018).

[3] Wright, S. L., Ulke, J., Font, A., Chan, K. L. & Kelly, F. J. Atmospheric microplastic deposition in an urban environment and an evaluation of transport. *Environ. Int*. **136**, 105411 (2020). 10.1016/j.envint.2019.105411 PMC7013824 31889555

[4] Cai L (2017). Characteristic of microplastics in the atmospheric fallout from Dongguan city, China: preliminary research and first evidence. Environ. Sci. Pollut. Res..

[5] Klein M, Fischer EK (2019). Microplastic abundance in atmospheric deposition within the Metropolitan area of Hamburg, Germany. Sci. Total Environ..

[6] Allen S (2019). Atmospheric transport and deposition of microplastics in a remote mountain catchment. Nat. Geosci..

[7] Brahney J, Hallerud M, Heim E, Hahnenbergere M, Sukumaran S (2020). Plastic rain in protected areas of the United States. Science.

[8] Brahney J (2021). Constraining the atmospheric limb of the plastic cycle. Proc. Natl Acad. Sci. USA.

[9] Wang X (2020). Atmospheric microplastic over the South China Sea and East Indian Ocean: abundance, distribution and source. J. Hazard. Mater..

[10] Liu K (2019). Consistent transport of terrestrial microplastics to the ocean through atmosphere. Environ. Sci. Technol..

[11] Bergmann, M., Mützel, S., Primpke, S., Tekman, M. B. Trachsel, J. & Gerdts, G. White and wonderful? Microplastics prevail in snow from the Alpss to the Arctic. *Sci. Adv*. **5**, eaxx1157 (2019). 10.1126/sciadv.aax1157 PMC6693909 31453336

[12] Durnford D, Dastoor A, Figueras-Nieto D, Ryjkov A (2010). Long range transport of mercury to the Arctic and across Canada. Atmos. Chem. Phys..

[13] Hirdman D (2009). Transport of mercury in the Arctic atmosphere: evidence for a springtime net sink and summer-time source. Geophys. Res. Lett..

[14] Evangeliou N (2020). Atmospheric transport, a major pathway of microplastics to remote regions. Nat. Commun..

[15] Uno I (2009). Asian dust transported one full circuit around the globe. Nat. Geosci..

[16] Marenco A, Gouget H, Nedelec P, Pages JP, Karcher F (1994). Evidence of a long-term increase in tropospheric ozone from Pic du Midi data series: consequences: positive radiative forcing. J. Geophys. Res..

[17] Fu X (2016). Atmospheric mercury speciation dynamics at the high-altitude Pic du Midi Observatory, southern France. Atmos. Chem. Phys..

[18] Gheusi F (2011). Pic 2005, a field campaign to investigate low-tropospheric ozone variability in the Pyrenees. Atmos. Res..

[19] Hulin M (2019). Observations of thermally driven circulations in the pyrenees: Comparison of detection methods and impact on atmospheric composition measured at a mountaintop. J. Appl. Meteorol. Climatol..

[20] Marusczak N, Sonke JE, Fu X, Jiskra M (2017). Tropospheric GOM at the Pic du Midi observatory-correcting bias in denuder based observations. Environ. Sci. Technol..

[21] Thinh, T. Q., Tran, T., Sang, N. & Viet, T. Q. Preliminary assessment on the microplastic contamination in the atmospheric fallout in the Phuoc Hiep landfill, Cu Chi, Ho Chi Minh city. *Vietnam J. Sci. Technol. Eng.* **62**, 83–89 (2020)..

[22] Cole M (2016). A novel method for preparing microplastic fibers. Sci. Rep..

[23] Kooi M, Koelmans AA (2019). Simplifying microplastic via continuous probability distributions for size, shape, and density. Environ. Sci. Technol. Lett..

[24] Draxler RR, Hess G (1998). D. An overview of the HYSPLIT_4 modelling system for trajectories, dispersion, and deposition. Aust. Meteorolofical Mag..

[25] Stein AF (2011). Modeling PM10 originating from dust intrusions in the Southern Iberian Peninsula using HYSPLIT. Weather Forecast.

[26] Suaria G (2020). Microfibers in oceanic surface waters: a global characterization. Sci. Adv..

[27] Babayemi, J. O., Nnorom, I. C., Osibanjo, O. & Weber, R. Ensuring sustainability in plastics use in Africa: consumption, waste generation, and projections. *Environ. Sci. Eur*. **31**, 1–20 (2019).

[28] Liss PS (2020). Microplastics: all up in the air?. Mar. Pollut. Bull..

[29] Li Y (2020). Airborne fiber particles: types, size and concentration observed in Beijing. Sci. Total Environ..

[30] Allen S (2020). Examination of the ocean as a source for atmospheric microplastics. PLoS ONE.

[31] Gaston, E., Woo, M., Steele, C., Sukumaran, S. & Anderson, S. Microplastics differ between indoor and outdoor air masses: insights from multiple microscopy methodologies. *Appl. Spectrosc*. 10.1177/0003702820920652 (2020). 10.1177/0003702820920652 32233850

[32] Dris R (2017). A first overview of textile fibers, including microplastics, in indoor and outdoor environments. Environ. Pollut..

[33] Lima, A. R. A. et al. Global patterns for the spatial distribution of floating microfibers: Arctic Ocean as a potential accumulation zone. *J. Hazard. Mater*. **403**, 123796 (2021). 10.1016/j.jhazmat.2020.123796 33264901

[34] Naik RA (2020). Microplastic particle versus fiber generation during photo-transformation in simulated seawater. Sci. Total Environ..

[35] Dommergue A (2019). Methods to investigate the global atmospheric microbiome. Front. Microbiol..

[36] Chevalier A (2008). Carbon monoxide observations from ground stations in France and Europe and long trends in the free troposphere. Atmos. Chem. Phys. Discuss.

[37] Ricaud P (2017). The GLAM airborne campaign across the Mediterranean Basin. Bull. Am. Meteorol. Soc..

[38] Henne S (2010). Assessment of parameters describing representativeness of air quality in-situ measurement sites. Atmos. Chem. Phys..

[39] Huppertsberg S, Knepper TP (2018). Instrumental analysis of microplastics—benefits and challenges. Anal. Bioanal. Chem..

[40] Menges, F. Spectragryph - optical spectroscopy software, Version 1.2.14, 2020. http://www.effemm2.de/spectragryph/ (2020).

[41] Munno K, Frond H, De, Donnell BO, Rochman CM (2020). Increasing the accessibility for characterizing microplastics: Introducing new application-based and spectral libraries of plastic particles (SLoPP & SLoPP-E). Anal. Chem..

[42] Schindelin J (2019). Fiji: an open-source platform for biological-image analysis. Nat. Methods.

[43] Wright, S. L., Levermore, J. M. & Kelly, F. J. Raman Spectral Imaging for the Detection of Inhalable Microplastics in Ambient Particulate Matter Samples. *Environ. Sci. Technol*. 10.1021/acs.est.8b06663 (2019). 10.1021/acs.est.8b06663 31293159

[44] Erni-cassola, G., Gibson, M. I., Thompson, R. C. & Christie-oleza, J. A. Lost, but found with Nile red; a novel method to detect and quantify small microplastics (20 µm–1 mm) in environmental samples. *Environ. Sci. Technol.* **51**, 13641–13648 (2017). 10.1021/acs.est.7b04512 29112813

[45] Stein AF (2015). NOAA’s HYSPLIT atmospheric transport and dispersion modeling system. Bull. Am. Meteorol. Soc..

[46] Pisso, I. et al. The Lagrangian particle dispersion model FLEXPART version 10.3. *Geosci. Model Dev. Discuss*. **12**, 4955–4997 (2019).

[47] Stohl A, Forster C, Frank A, Seibert P, Wotawa G (2005). Technical note: the Lagrangian particle dispersion model FLEXPART version 6.2. Atmos. Chem. Phys. Discuss..

[48] Stohl, A. et al. The Lagrangian particle dispersion model FLEXPART version 9.3. https://www.flexpart.eu/downloads/54 (2016).

[49] Seibert P, Frank A (2004). Source-receptor matrix calculation with a Lagrangian particle dispersion model in backward mode. Atmos. Chem. Phys..

[50] Henn AR (1996). Calculation of the stokes and aerodynamic equivalent diameters of a short reinforcing fiber. Part. Part. Syst. Charact..

[51] Liu K (2019). Source and potential risk assessment of suspended atmospheric microplastics in Shanghai. Sci. Total Environ..

[52] Yurtsever, M., Kaya, A. & Bayraktar, C. A research on Microplastic Presence in Outdoor Air. In *International Conference on Microplastic Pollution in the Mediterranean Sea* (ed. Cocca, M.) Vol. 22, 238 (Springer International Publishing, 2018).

[53] Kaya A, Yurtsever M, Bayraktar S (2018). Ubiquitous exposure to microfiber pollution in the air. Eur. Phys. J..

[54] Asrin N, Dipareza A (2019). Microplastics in ambient air (case study: Urip Sumoharjo Street and Mayjend Sungkono Street of Surabaya City, Indonesia). IAETSD J. Adv. Res. Appl. Sci..

[55] Abbasi S (2019). Distribution and potential health impacts of microplastics and microrubbers in air and street dusts from Asaluyeh County, Iran. Environ. Pollut..

[56] Akhbarizadeh, R., Dobaradaran, S., Torkmahalleh, M. A., Saeedi, R. & Ghasemi, F. F. Suspended fine particulate matter (PM2.5), microplastics (MPs), and polycyclic aromatic hydrocarbons (PAHs) in air: their possible relationships and health implications. *Environ. Res*. 116544 10.1016/j.envres.2020.110339 (2020). 10.1016/j.envres.2020.110339 33068583

