# Supplementary material for: Evidence of free tropospheric and long-range transport of microplastic at Pic du Midi Observatory
Source: Nat Commun. 2021 Dec 21;12:7242. doi: 10.1038/s41467-021-27454-7 (PMC8692471; doi:10.1038/s41467-021-27454-7)
Supplement: Supplementary file 1 — Supplementary Information [file 41467_2021_27454_MOESM1_ESM.docx]

Supplementary Figures for ‘Evidence of free tropospheric and long-range transport of microplastic at Pic du Midi Observatory’ Allen et al.

Supplementary Figure 1. Individual sample period air mass particle history modelled trajectory elevations and durations.


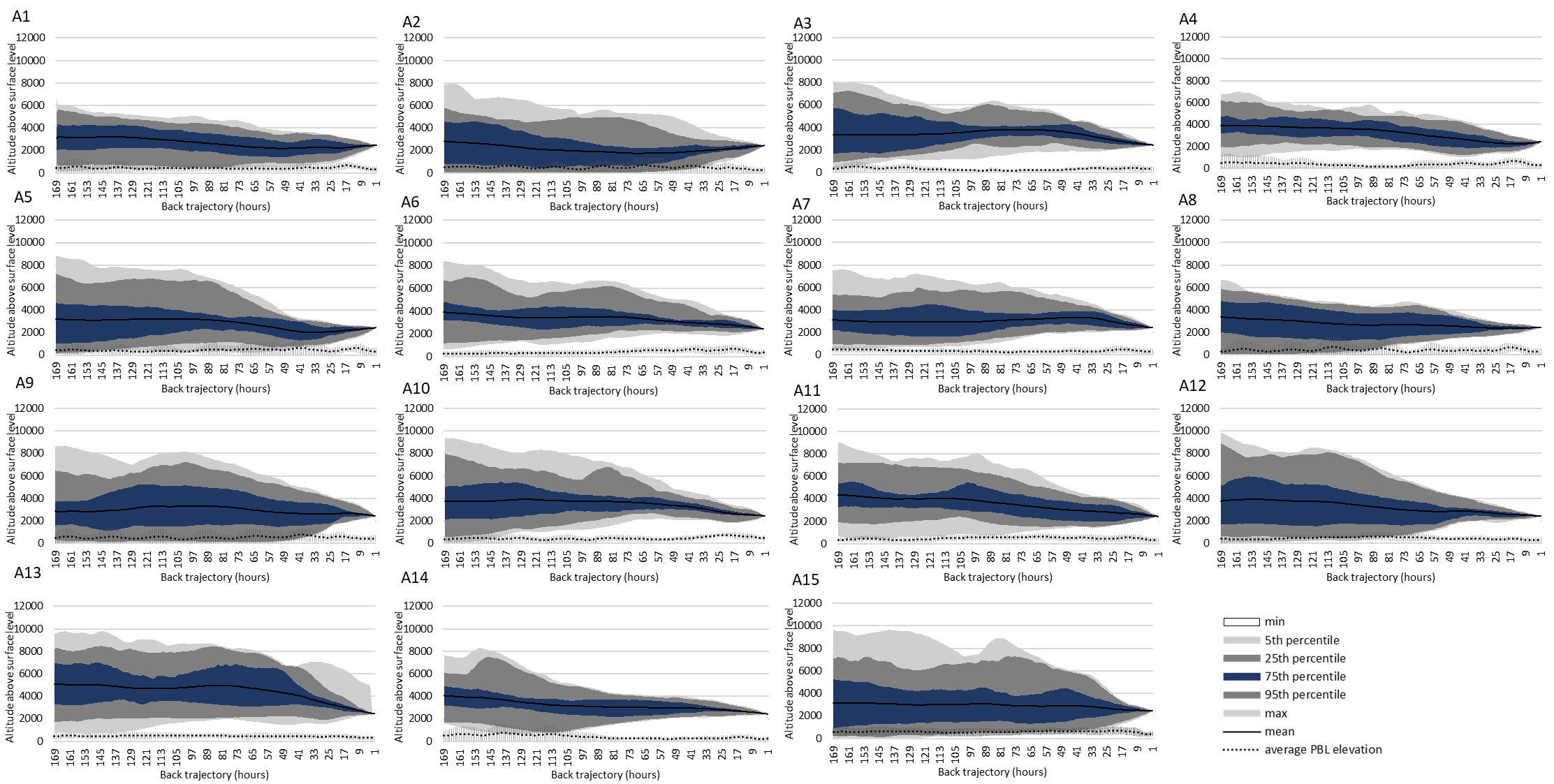


Supplementary Figure 2. Individual sample period modelled back trajectory extents and elevations above surface level.


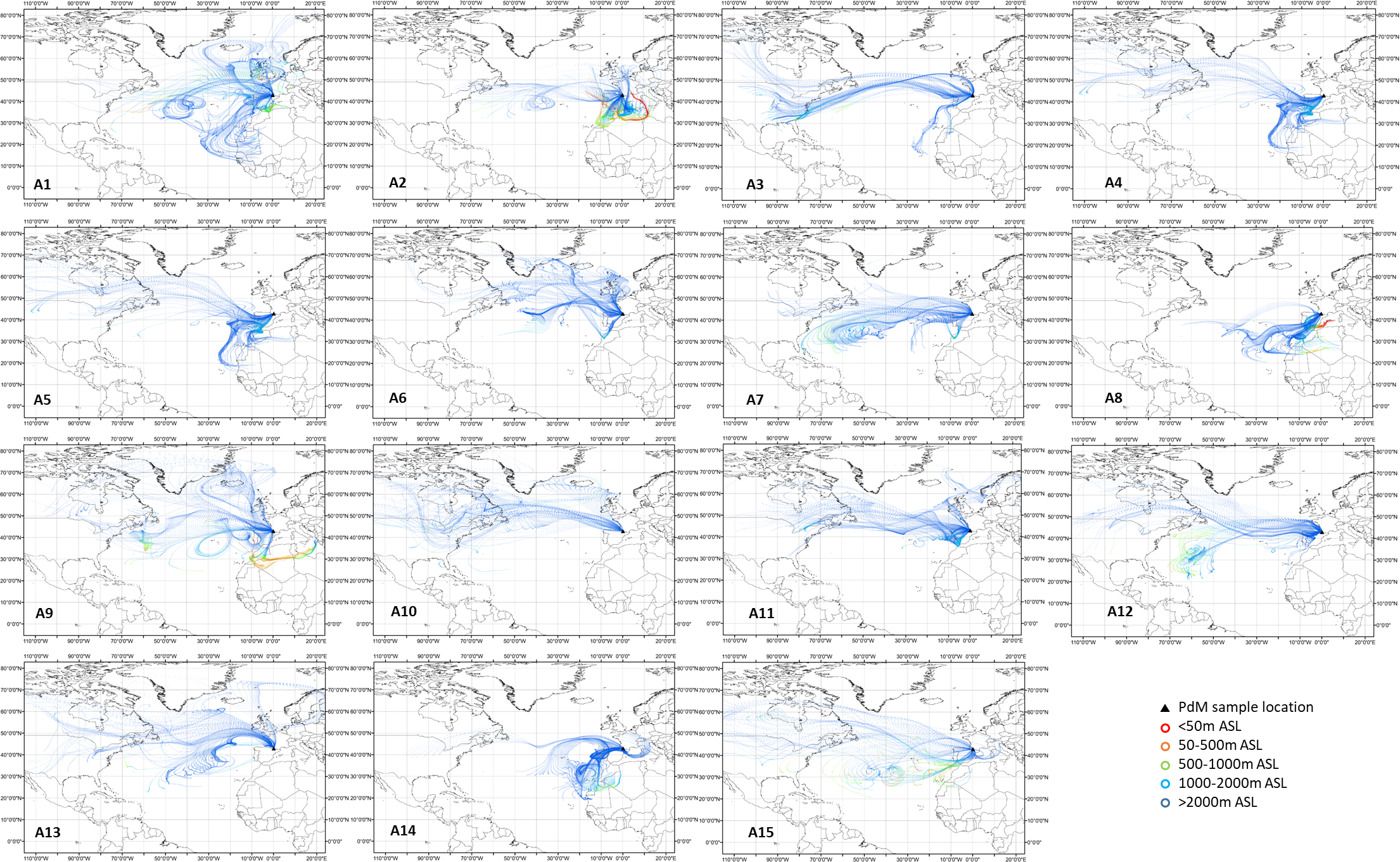


Supplementary Figure 3. Individual sample period back trajectory points within the PBL in elevations surface level.


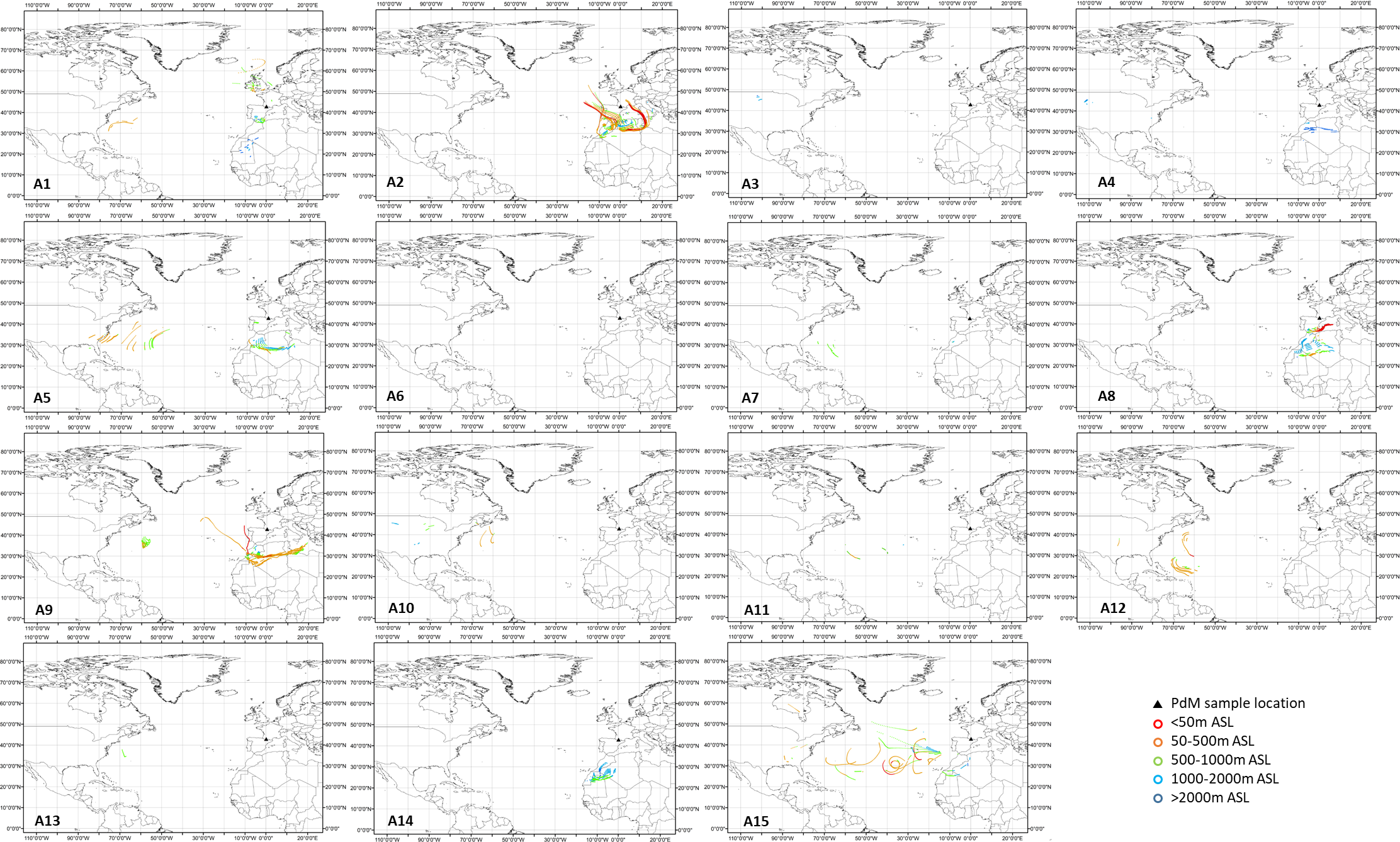


Supplementary Figure 4. 25^th^ Percentile MP comparative dataset


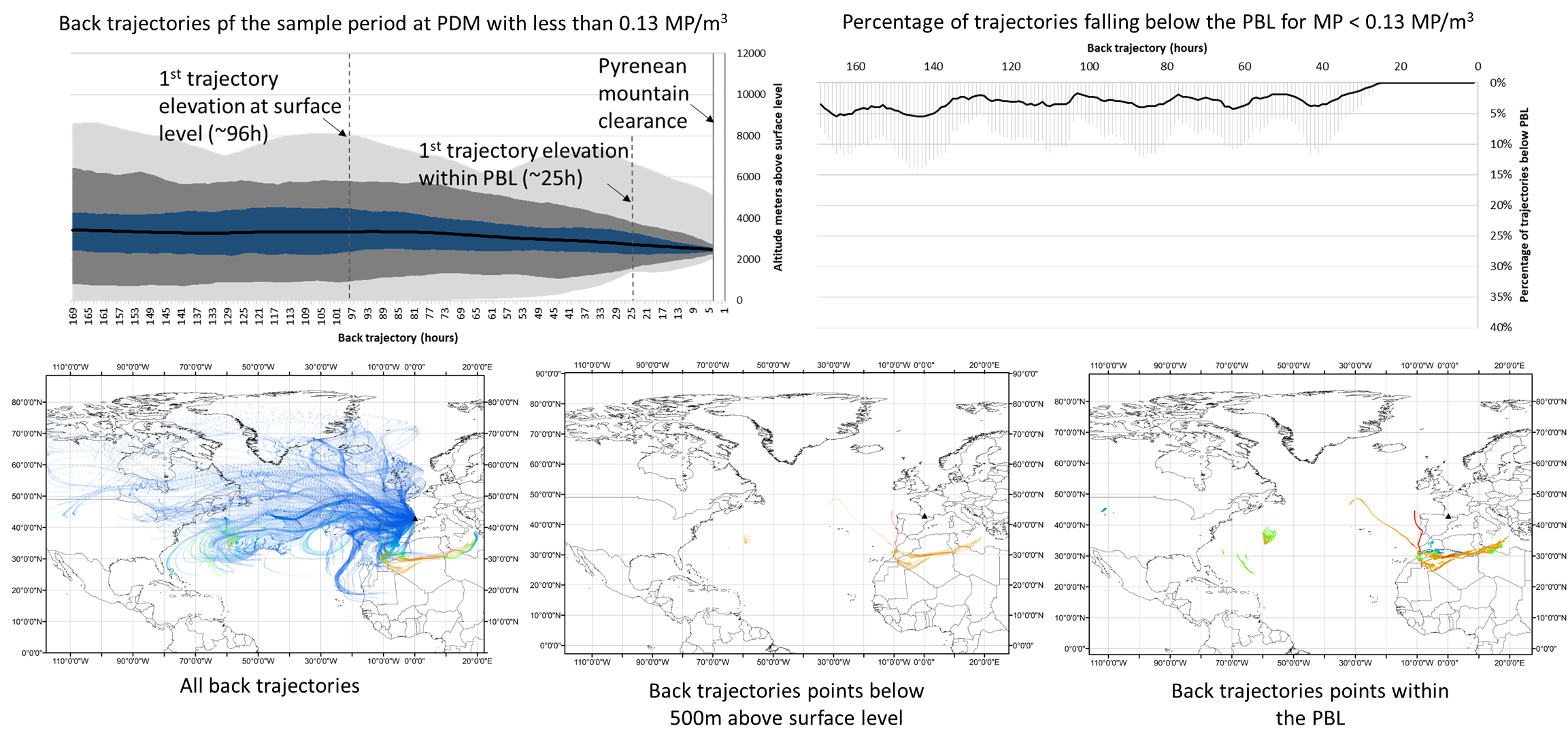


The 25^th^ percentile of PDM MP includes samples A4, A6, A7 and A9 (MP < 0.13 MP/m^3^). While there are some differentiations identifiable between 0.33 < MP > 0.33 MP/m^3^ (the 75^th^ percentile of PDM MP samples) this is less obvious for the lower MP samples. Only one (A9) sample in this lower quartile has backward modelling of trajectories at surface level (0m ASL), and three of the samples (A4, A7 and A9) include modelled back trajectories with PBL/FT mixing. However, the closest proximity to PDM of this PBL/FT fixing occurring is notably greater (887 km) than for the MP < 0.33 MP/m^3^ (350 km).

Supplementary Figure 5. Comparison of the microplastic particle counts per sample (MP/m^3^) to the modelled atmospheric back trajectories for each sample period that fell below the PBL during the 168 hour back trajectory modelling duration.

(b)

(a)

There is also a positive correlation between the number of trajectories in the PBL and the MP count in the air mass and the quantity of MP >10µm (Fig S5b: r = 0.80, p value < 0.05 (r2 = 0.62) respectively). This is also evident in the comparison of MP counts to the percentage of trajectories within the PBL (MP counts (all) r = 0.69, p < 0.05; MP counts >10µm r = 0.78, p < 0.05). This indicates that frequency of the influence of the PBL may directly impact the MP concentration in the PDM air mass, with a greater MP concentration when a higher proportion of the contributing air masses pass through the PBL within the prior 186 hours.

When the sample specific back trajectory duration within the PBL is compared to the atmospheric MP count at PDM there is a visual positive trend but a non-significant low correlation (r=0.48, p>0.05). This suggests that for this sample location and duration the frequency of trajectory transit through the PBL appears to potentially be of greater influence that the duration of time trajectories spend within the PBL overall. However, further field sampling and extended duration assessment is necessary to explore this potential correlation and influence in detail.

The number of PBL mixing points occurring over land or sea appears to correlate with the total MP counts and MP > 10µm. This supports the correlation and potential importance of the PBL/FT mixing frequency on MP content in the PDM air samples for this duration, but does not illustrate a preference or differentiation between land or sea PBL/FT mixing locations relative to MP sample quantities. This tentatively concurs with modelling undertaken for the USA and globally^1^.

Supplementary Figure 6. Summary of published atmospheric microplastic findings. The table includes both pumped air monitoring results and passive deposition, whereas Table 1 of the manuscript focuses on only actively pumped sample findings for a more direct comparison.

| Location | Microplastic count | Microplastic mean or median counts | MP size range | Predominant size ranges | Environment | Reference |
| --- | --- | --- | --- | --- | --- | --- |
| Dongguan, China | 175-313 MP/day/m^2^ | 228 MP/day/m^2^ | 200µm-5mm | 200-700µm | City | ^2^ |
| Shanghai, China | 0-4.18 MP/m^3^ | 1.42 MP/m^3^ | 23µm-5mm | <1000µm | City | ^3^ |
| Yantai, China | 0-602 MP/day/m^2^ | NA | 100µm-5mm | 100-300µm | City | ^4^ |
| China cities (39) | 2-9020 mg/kg | 2780mg/kg PET 2mg/kg PC | NA | NA | City | ^5^ |
| Paris, France | 2-355 MP/day/m^2^ | 110 MP/day/m^2^ | 50µm-5mm | 200-400µm | City | ^6^ |
| Paris (indoor) | 0.4-60.0 MP/m^3^ | 5.4 MP/m^3^ | 50µm-5mm | 20-250µm | City | ^7^ |
|  | 1586-11,130 MP/day/m^2^ | 6200 MP/day/m^2^ |  | NA |  |  |
| Paris (outdoor) | 0.3-1.5 MP/m^3^ | 0.9 MP/m^3^ | 50µm-5mm | 20-250µm | City | ^7^ |
| Shanghai, China (indoor) | 500 -24000 MP/day/m^2^ | 5880 MP/day/m^2^ | 50-5000µm | <2000µm | Indoor City | ^8^ |
| Sakarya Province, Turkey | 116-3424 MP/m^3^ | 2019 MP/m^3^ | 50µm-5mm | NA | City | ^9,10^ |
| Pyrenees Mountains, France | 204-599 MP/day/m^2^ | 365 MP/day/m^2^ | 10µm-5mm | 10-25µm | Remote terrestrial (mountains) | ^11^ |
| Hamburg, Germany | 25-800 MP/day/m^2^ | 428 MP/day/m^2^ forest 331 MP/day/m^2^ rural 215 MP/day/m^2^ city | 63µm-5mm | <63µm fragments 300-5000µm fibres | City, Rural, Forest | ^12^ |
| Arctic snow | 0-14.4x10^3^ MP/L | 1.4x10^3^ MP/L | 11µm-5mm | 100-200µm and 900-1000µm fibres <25µm fragments | Remote Arctic | ^13^ |
| European snow | 0.2-154 x10^3^ MP/L | 25x10^3^ MP/L | 11µm-5mm | 800-1000µm fibres <25µm fragments | Remote terrestrial (mountains) and Arctic | ^13^ |
| Italian Alps | 37-137 MP/kg | 74 MP/kg | NA | NA | Remote terrestrial (mountains) | ^14^ |
| Helsinki, Finland | 84-5935 MP/m^3^ | 1762 MP/m^3^ | 0.3-4mm | 0.3-1mm | Urban snow | ^15^ |
| Western Pacific Ocean (Shanghai - Mariana Islands) | 0-1.37 MP/m^3^ | 0.01 MP/m^3^ | 20µm-2mm | 318µm | Offshore marine air | ^16^ |
| Surabaya, Indonesia | 131-174 MP/m^3^ | 153 MP/m^3^ | ~500µm-5mm | 1000-1500µm | City | ^17^ |
| Asaluyeh County, Iran | 0.3-1.1 MP/m^3^ | NA | 100µm-1mm | >100µm fibres | City | ^18^ |
| Beijing, China | 5600-5700 MP/m^3^ | 5600 MP/m^3^ | 5µm – 2mm | <20µm | City | ^19^ |
| Tehran, Iran | 88-605 MP/30g dust | 276 MP/30g dust | 100µm-1mm | 250-500µm | City | ^20^ |
| Nottingham, UK | 0-31 MP/day/m^2^ | 2.9 MP/day/m^2^ fibres | 38µm-5mm | NA | City and urbanised area | ^21^ |
| Atlantic coast, France | 0.02-19 MP/m^3^ | 2.9 MP/m^3^ (onshore) | 2.5-300µm | 5-10µm | Coastal onshore air | ^22^ |
| London, UK | 575-1008 MP/day/m^2^ | 712 MP/day/m^2^ fibres 59 MP/day/m^2^ fragments | 25µm-3000µm | 400-500µm fibres 164µm fragments | City | ^23^ |
| Cal State University, USA (indoor) | 3-17 MP/m^3^ fibres  2.7-8.3 MP/m^3^ fragments | 9.8MP/m^3^ fibres  6.7 MP/m^3^ fragments | 20->3000µm | 100-300µm fibre | City | ^24^ |
| Cal State University, USA (outdoor) | 0.7-2.2 MP/m^3^ fibres  12.5-19.6 MP/m^3^ fragments | 1.5 MP/m^3^ fibres  15.5 MP/m^3^ fragments | 20->3000µm | 100-300µm fibre | City | ^24^ |
| Pearl River Estuary, South China Sea, Indian Ocean | 0-0.077 MP/m^3^ | 0.01 MP/m^3^ | 58-2252µm | 851µm | Offshore marine air | ^25^ |
| North America wilderness areas | 48-435 MP/day/m^2^ | 132 MP/day/m^2^ | 4-3000µm | <25µm | Remote terrestrial (national parks) | ^26^ |
| Bushehr port, Iran | 0-14.2 MP/m^3^ | 2.1 MP/m^3^ calm days  10.3 MP/m^3^ dusty days | <2.5µm | NA | City | ^27^ |
| Sonnblick Observatory, Austria | 4.6-23.6 ng/mL | 12.9 ng/mL | 0.2-1µm | <1µm | Remote terrestrial (snow) | ^28^ |
| Sonnblick Observatory, Austria | 0-68 ng/ml/day | 46.5 ng/mL (nanoplastics) | <1µm | <1µm | Remote terrestrial (snow) | ^29^ |
| Ireland, EU | 64-102 MP/day/m^2^ | 80 MP/m^2^/day | 50µm-5mm (0.04-19.75mm length) | fibres | Rural outdoor deposition | ^30^ |
| Ho Chi Minh city landfill, Vietnam | 427-2444MP/day/m^2^ | 1801 MP/m^2^ dry 913 MP/m^2^ wet | 100-5000-µm | 100-300µm | landfill | ^31^ |
| Antisana glacier, Ecuadorian Andes | 270 MP/250 snow 89.7MP/10x10m2 | 0.9 MP/m^2^ | 60-2500µm | fibres only | glacial ice | ^32^ |
| Alert, Nunavut | 0-4 MP/450ml | NA | 20µm-5mm | NA | Snow | ^33^ |
| Mt Everest | 3-119 MP/L | 30 MP/L | 18-5000µm | 36-3800µm length 10-2000µm diameter | Snow/glacier | ^34^ |
| Pic du Midi, France | 0.09-0.66 MP/m^3^ | 0.23 MP/m^3^ | 5-163µm | <10µm fragments 15-20µm fibres | Remote terrestrial (mountains) | This Study |

References

1. Brahney, J. *et al.* Constraining the atmospheric limb of the plastic cycle. *PNAS* **118**, e2020719118 (2021).

2. Cai, L. *et al.* Characteristic of microplastics in the atmospheric fallout from Dongguan city, China: preliminary research and first evidence. *Environ. Sci. Pollut. Res.* **24**, 24928–24935 (2017).

3. Liu, K. *et al.* Source and potential risk assessment of suspended atmospheric microplastics in Shanghai. *Sci. Total Environ.* **675**, 462–471 (2019).

4. Zhou, Q., Tian, C. & Luo, Y. Various forms and deposition fluxes of microplastics identified in the coastal urban atmosphere. *Chinese Sci. Bull.* **62**, 3902–3909 (2017).

5. Liu, C. *et al.* Widespread distribution of PET and PC microplastics in dust in urban China and their estimated human exposure. *Environ. Int.* **128**, 116–124 (2019).

6. Dris, R., Gasperi, J., Saad, M., Mirande, C. & Tassin, B. Synthetic fibers in atmospheric fallout: A source of microplastics in the environment? *Mar. Pollut. Bull.* **104**, 290–293 (2016).

7. Dris, R. *et al.* A first overview of textile fibers, including microplastics, in indoor and outdoor environments. *Environ. Pollut.* **221**, 453–458 (2017).

8. Zhang, Q. *et al.* Microplastic Fallout in Different Indoor Environments. *Environ. Sci. Technol.* 0–32 (2020) doi:10.1021/acs.est.0c00087.

9. Kaya, A., Yurtsever, M. & Bayraktar, S. Ubiquitous exposure to microfiber pollution in the air. *Eur. Phys. J. Plus* **133**, 1–9 (2018).

10. Yurtsever, M., Kaya, A. & Bayraktar, C. A research on Microplastic Presence in Outdoor Air. in *International Conference on Microplastic Pollution in the Mediterranean Sea* (ed. Cocca, M.) vol. 22 238 (Springer International Publishing, 2018).

11. Allen, S. *et al.* Atmospheric transport and deposition of microplastics in a remote mountain catchment. *Nat. Geosci.* **12**, 339–344 (2019).

12. Klein, M. & Fischer, E. K. Microplastic abundance in atmospheric deposition within the Metropolitan area of Hamburg, Germany. *Sci. Total Environ.* **685**, 96–103 (2019).

13. Bergmann, M. *et al.* White and wonderful? Microplastics prevail in snow from the Alps to the Arctic. *Sci. Adv.* **5**, eaax1157 (2019).

14. Ambrosini, R. *et al.* First evidence of microplastic contamination in the supraglacial debris of an alpine glacier. *Environ. Pollut.* **253**, 297–301 (2019).

15. Pikkarainen, K. Pure as snow? Snow as a route for microplastics and other waste from urban areas to sea. (Universiyt of Helsinki, 2017).

16. Liu, K. *et al.* Consistent transport of terrestrial microplastics to the ocean through atmosphere. *Environ. Sci. Technol.* **53**, 10612–10619 (2019).

17. Asrin, N. & Dipareza, A. Microplastics in Ambient Air (Case Study : Urip Sumoharjo Street and Mayjend Sungkono Street of Surabaya City , Indonesia). *IAETSD J. Adv. Res. Appl. Sci.* **6**, 54–57 (2019).

18. Abbasi, S. *et al.* Distribution and potential health impacts of microplastics and microrubbers in air and street dusts from Asaluyeh County, Iran. *Environ. Pollut.* **244**, 153–164 (2019).

19. Li, Y. *et al.* Airborne fiber particles: Types, size and concentration observed in Beijing. *Sci. Total Environ.* **705**, 135967 (2020).

20. Dehghani, S., Moore, F. & Akhbarizadeh, R. Microplastic pollution in deposited urban dust, Tehran metropolis, Iran. *Environ. Sci. Pollut. Res.* **24**, 20360–20371 (2017).

21. Stanton, T., Johnson, M., Nathanail, P., MacNaughtan, W. & Gomes, R. L. Freshwater and airborne textile fibre populations are dominated by ‘natural’, not microplastic, fibres. *Sci. Total Environ.* **666**, 377–389 (2019).

22. Allen, S., Allen, D., Moss, Kerry Le Roux, G., Phoenix, V. R. & Sonke, J. Examination of the ocean as a source for atmospheric microplastics. *PLoS One* **2018**, 1–14 (2020).

23. Wright, S. L., Ulke, J., Font, A., Chan, K. L. . & Kelly, F. J. Atmospheric microplastic deposition in an urban environment and an evaluation of transport. *Environ. Int.* **136**, (2020).

24. Gaston, E., Woo, M., Steele, C., Sukumaran, S. & Anderson, S. Microplastics Differ Between Indoor and Outdoor Air Masses : Insights from Multiple Microscopy Methodologies. *Appl. Spectrosc.* (2020) doi:10.1177/0003702820920652.

25. Wang, X. *et al.* Atmospheric microplastic over the South China Sea and East Indian Ocean : abundance , distribution and source. *J. Hazard. Mater.* **389**, 121846 (2020).

26. Brahney, J., Hallerud, M., Heim, E., Hahnenbergere, M. & Sukumaran, S. Plastic rain in protected areas of the United States. *Science (80-. ).* **368**, 1257–1260 (2020).

27. Akhbarizadeh, R., Dobaradaran, S., Torkmahalleh, M. A., Saeedi, R. & Ghasemi, F. F. Suspended fine particulate matter (PM2.5), microplastics (MPs), and polycyclic aromatic hydrocarbons (PAHs) in air: Their possible relationships and health implications. *Environ. Res.* 116544 (2020) doi:10.1016/j.envres.2020.110339.

28. Materić, D. *et al.* Micro- and nanoplastics in Alpine snow – a new method for chemical identification and quantification in the nanogram range. *Environ. Sci. Technol.* **54**, 2353–2359 (2020).

29. Materić, D., Ludewig, E., Brunner, D., Rochmann, T. & Holzinger, R. Nanoplastics transport to the remote, high-altitude Alps. *Environ. Pollut.* **288**, (2021).

30. Roblin, B., Ryan, M., Vreugdenhil, A. J. & Aherne, J. Ambient atmospheric deposition of anthropogenic microfibres and microplastics on the western periphery of Europe ( Ireland ). *Environ. Sci. Technol.* (2020) doi:10.1021/acs.est.0c04000.

31. Thinh, T. Q., Tran, T., Sang, N. & Viet, T. Q. Preliminary assessment on the microplastic contamination in the atmospheric fallout in the Phuoc Hiep landfill , Cu Chi , Ho Chi Minh city. *Vietnam J. Sci. Technol. Eng.* **62**, 83–89 (2020).

32. Cabrera, M. *et al.* A new method for microplastic sampling and isolation in mountain glaciers: A case study of one antisana glacier, Ecuadorian Andes. *Case Stud. Chem. Environ. Eng.* **2**, 100051 (2020).

33. Huntington, A. *et al.* A first assessment of microplastics and other anthropogenic particles in Hudson Bay and the surrounding eastern Canadian Arctic waters of Nunavut. *Facets* **5**, 432–454 (2020).

34. Napper, I. E. *et al.* Reaching New Heights in Plastic Pollution — Preliminary Findings of Microplastics on Mount Everest Reaching New Heights in Plastic Pollution — Preliminary Findings of Microplastics on Mount Everest. *One Earth* **3**, 621–630 (2020).
