# Supplementary material for: Evidence of free tropospheric and long-range transport of microplastic at Pic du Midi Observatory
Source: Nat Commun. 2021 Dec 21;12:7242. doi: 10.1038/s41467-021-27454-7 (PMC8692471; doi:10.1038/s41467-021-27454-7)
Supplement: Supplementary file 2 — Description of Additional Supplementary Files [file 41467_2021_27454_MOESM2_ESM.docx]

Description of Additional Supplementary Files

File name: Supplementary Data 1

Description: The supplementary file includes the figures referred to in the main test, including supporting dataset of MP counts, MP characteristics and Local Meteorology relative to each sampling period. Specifically, the following figures are presented:

Supplementary Figure 1. Individual sample period air mass history modelled trajectory elevations and durations

Supplementary Figure 2. Individual sample period modelled back trajectory extents and elevations above surface level

Supplementary Figure 3. Individual sample period back trajectory points within the PBL in elevations surface level

Supplementary Figure 4. 25th Percentile MP comparative dataset

Supplementary Figure 5. Comparison of the microplastic particle counts per sample (MP/m3) to the modelled atmospheric back trajectories for each sample period that fell below the PBL during the 168 hour back trajectory modelling duration

Supplementary Figure 6. Summary of published atmospheric microplastic findings.
