# Supplementary material for: Evidence of free tropospheric and long-range transport of microplastic at Pic du Midi Observatory
Source: Nat Commun. 2021 Dec 21;12:7242. doi: 10.1038/s41467-021-27454-7 (PMC8692471; doi:10.1038/s41467-021-27454-7)
Supplement: Supplementary file 4 — Reporting Summary [file 41467_2021_27454_MOESM4_ESM.pdf]

nature portfolio

Corresponding author(s): Steve Allen

Last updated by author(s): Oct 18, 2021

Reporting Summary

Nature Portfolio wishes to improve the reproducibility of the work that we publish. This form provides structure for consistency and transparency in reporting. For further information on Nature Portfolio policies, see our Editorial Policies and the Editorial Policy Checklist.

Statistics

For all statistical analyses, confirm that the following items are present in the figure legend, table legend, main text, or Methods section.

n/a	Confirmed	
☐	☒	The exact sample size (n) for each experimental group/condition, given as a discrete number and unit of measurement
☐	☒	A statement on whether measurements were taken from distinct samples or whether the same sample was measured repeatedly
☒	☐	The statistical test(s) used AND whether they are one- or two-sided<br>*Only common tests should be described solely by name; describe more complex techniques in the Methods section.*
☒	☐	A description of all covariates tested
☐	☒	A description of any assumptions or corrections, such as tests of normality and adjustment for multiple comparisons
☐	☒	A full description of the statistical parameters including central tendency (e.g. means) or other basic estimates (e.g. regression coefficient) AND variation (e.g. standard deviation) or associated estimates of uncertainty (e.g. confidence intervals)
☒	☐	For null hypothesis testing, the test statistic (e.g. F, t, r) with confidence intervals, effect sizes, degrees of freedom and P value noted<br>*Give P values as exact values whenever suitable.*
☒	☐	For Bayesian analysis, information on the choice of priors and Markov chain Monte Carlo settings
☒	☐	For hierarchical and complex designs, identification of the appropriate level for tests and full reporting of outcomes
☐	☒	Estimates of effect sizes (e.g. Cohen's d, Pearson's r), indicating how they were calculated

Our web collection on statistics for biologists contains articles on many of the points above.

Software and code

Policy information about availability of computer code

Data collection	none used
Data analysis	Data was analysed using Spectragryph 1.2.14 to manage Raman spectra (F. Menges "Spectragryph - optical spectroscopy software", Version 1.x.x, 201x, http://www.effemm2.de/spectragryph/), FIJI/Image J for particle size characterisation and analysis (Schindelin, J., Arganda-Carreras, I., Frise, E., Kaynig, V., Longair, M., Pietzsch, T., … Cardona, A. (2012). Fiji: an open-source platform for biological-image analysis. Nature Methods, 9(7), 676–682. doi:10.1038/nmeth.2019), in accordance with the methods detailed in Allen et al. 2019. Raman spectra was analysed using the publically available SLOPP and SLOPPE libraries and in-house validation polymer tests (Munno, K., Frond, H. De, Donnell, B. O. & Rochman, C. M. Increasing the accessibility for characterizing microplastics : Introducing new application-based and spectral libraries of plastic particles (SLoPP & SLoPP-E). Anal. Chem. 92, 2443–2451 (2020)). Atmospheric modelling was completed using HYSPLIT version 4 and FLEXPART version 9.02 (Draxler, R. R. & Hess, G, D. An Overview of the HYSPLIT_4 Modelling System for Trajectories, Dispersion, and Deposition. Aust. Meteorolofical Mag. 47, 295–308 (1998); Seibert, P. & Frank, A. Source-receptor matrix calculation with a Lagrangian particle dispersion model in backward mode. Atmos. Chem. Phys. 4, 51–63 (2004)). Mapping software ArcMap 10.4 was used to help plot the Hysplit outputs.

For manuscripts utilizing custom algorithms or software that are central to the research but not yet described in published literature, software must be made available to editors and reviewers. We strongly encourage code deposition in a community repository (e.g. GitHub). See the Nature Portfolio guidelines for submitting code & software for further information.

Data

Policy information about availability of data

All manuscripts must include a data availability statement. This statement should provide the following information, where applicable:

- Accession codes, unique identifiers, or web links for publicly available datasets
- A description of any restrictions on data availability
- For clinical datasets or third party data, please ensure that the statement adheres to our policy

The microplastic data generated in this study has been provided in the Supplementary dataset (xls) provided with this manuscript. All data needed to evaluate the conclusion in the paper are present in the paper and/or supplementary materials. All samples, analysis, publication and ownership of data are free from legal entanglement or restriction of any sort. Meteorological datasets of the Pyrenean Platform for Observation of the Atmosphere (P2OA) can be accessed online (http://p2oa.aero.obs-mip.fr).

Field-specific reporting

Please select the one below that is the best fit for your research. If you are not sure, read the appropriate sections before making your selection.

☐ Life sciences ☐ Behavioural & social sciences ☒ Ecological, evolutionary & environmental sciences

For a reference copy of the document with all sections, see nature.com/documents/nr-reporting-summary-flat.pdf

Ecological, evolutionary & environmental sciences study design

All studies must disclose on these points even when the disclosure is negative.

Study description	The study was designed to collect and quantitatively characterise microplastic from the high altitude, 'remote', long term observation site Pic du Midi Biggore in the French Pyrenees mountains. The study quantified the microplastic concentrations in all samples at this high altitude station (collected summer/autumn of 2017, 23/06/2017-23/10/2017, 15 samples collected in triplicate). Analysis of the atmospheric transport of the microplastic particles was then undertaken to establish the elevation (altitude) of atmospheric transport to the field site and the transport path (distance, spatial path) that these particles may have followed. The atmospheric transport model outputs (spatial pathways and elevations) were then compared to the microplastic concentrations to identify any trend between the regional and long distance 'flight path' of the particles relative to the quantity of microplastic collected in each sample.
Research sample	Samples are atmospheric active (pumped air) samples collected using a standard TISCH HiVol system, placed outdoor to collect remote area atmospheric air samples at the Pic du Midi Biggore long term observation field site.
Sampling strategy	The study endeavored to determine the presence and quantity of atmospheric microplastics and free tropospheric transport. The duration and number of samples was chosen to ensure sufficient data points for statistical validly (n>3). The 15-week sample set, samples available for microplastic analysis to this study, were considered to provide sufficient repetition (n>>3) to evidence free tropospheric microplastic transport.<br>Samples were collected using a TISCH HiVol atmospheric sampling system, collected or quartz filters with a pore size of 2.2µm. Samples were transported to the laboratory where triplicate microplastic samples were taken. Samples were then transferred from the quartz filters to aluminium oxide filters (25mm dia., 0.2µm pore size) to Raman analysis.
Data collection	µRaman spectroscopic analysis of 30% of the filter area was completed to identify the polymer composition and microplastic count for each sample. To confirm these counts visual microscopy was used. The Limit of quantification was set to 2.2µm. Raman spectra were analysed using existing published polymer libraries to identify polymer type. Image J was used to identify particle size and shape. All data was created, collated and analysed by S Allen and D Allen.
Timing and spatial scale	Sampling occurred over the summer/autumn of 2017, 23/06/2017-23/10/2017. These are the seasons most likely to be influenced by convection and elevated planetary boundary layer.
Data exclusions	no data was excluded from this study
Reproducibility	All samples were taken in triplicate to support reproducibility. The atmospheric samples is a standard piece of equipment commonly used in atmospheric measurements and previously validated for atmospheric particle sampling, with published protocols for sample collection. The Raman analysis follows previously published protocols that have been used by multiple researchers, reproducible through methods detail published to date. All software used is open source and available to all researchers.
Randomization	Randomisation in the sample collections is not relevant to this study as the study is designed as an analysis of atmospheric microplastic concentration specific to this location over this specified duration. The grouping used in the final analysis (MP greater or lesser than 0.33MP/m3) was defined through statistical calculation of the upper 25th percentile, thus the higher concentration within the sample dataset specific to individual samples.
Blinding	Blinding was not incorporated into this study. All data has been considered in the sampling, analysis and discussion of this study to ensure bias and blinding is prevented as far as possible.

Did the study involve field work? ☒ Yes ☐ No

Field work, collection and transport

Field conditions	The field work collected atmospheric microplastics from pumped air samples. Meteorological observations were not completed specifically for this study but the available meteorology data (rainfall, wind velocity direction, temperature etc.) form part of the Pyrenean Platform for Observation of the Atmosphere (P2OA) can be accessed online (http://p2oa.aero.obs-mip.fr).
Location	Samples were collected from the HiVol air sampling equipment installed for long term monitoring at the Pic du Midi Biggore high altitude field observation station, 42°56′11″N 0°08′34″E, 2877 m above mean sea level.
Access & import/export	The samples are aerosol particles and therefore required no import/export permits or licenses. The samples were collected from the field site and transported in airtight sealed containers to the laboratory (vehicular transport). The access to the monitoring facility, sampling collection and transport of these aerosol filters was in full compliance with local and national requirements.
Disturbance	No disturbance was caused. In situ measurement systems were used to ensure minimal disturbance.

Reporting for specific materials, systems and methods

We require information from authors about some types of materials, experimental systems and methods used in many studies. Here, indicate whether each material, system or method listed is relevant to your study. If you are not sure if a list item applies to your research, read the appropriate section before selecting a response.

Materials & experimental systems

n/a	Involved in the study
☒	☐ Antibodies
☒	☐ Eukaryotic cell lines
☒	☐ Palaeontology and archaeology
☒	☐ Animals and other organisms
☒	☐ Human research participants
☒	☐ Clinical data
☒	☐ Dual use research of concern

Methods

n/a	Involved in the study
☒	☐ ChIP-seq
☒	☐ Flow cytometry
☒	☐ MRI-based neuroimaging
